# Textbook oncologic outcomes after cytoreductive surgery and HIPEC for colorectal peritoneal metastases: a single-centre cohort analysis

**DOI:** 10.1515/pp-2025-0038

**Published:** 2026-04-16

**Authors:** Niall Brindl, Arianna Castagna, Thilo Welsch, Mohammed Al-Saeedi, Martin Schneider, Markus Büchler, Andreas Brandl

**Affiliations:** Department of General, 27178 Visceral and Transplantation Surgery, University Hospital Heidelberg, Heidelberg, Germany; Department for General, Visceral and Tumour Surgery, Krankenhaus Nordwest, Frankfurt/Main, Germany; Department of General, Visceral, Thoracic and Transplantation Surgery, University Hospital Giessen, Giessen, Germany; Botton-Champalimaud Pancreatic Cancer Center, Champalimaud Foundation, Lisboa, Portugal

**Keywords:** textbook oncological outcome, colorectal cancer, cytoreductive surgery, hyperthermic intraperitoneal chemotherapy

## Abstract

**Objectives:**

Cytoreductive surgery (CRS) combined with hyperthermic intraperitoneal chemotherapy (HIPEC) is an established treatment for selected patients with colorectal peritoneal metastases (CPM). The recently proposed concept of textbook oncologic outcomes (TOO) offers a composite benchmark for surgical quality, but its applicability and prognostic relevance in CPM remain largely unexplored.

**Methods:**

We conducted a retrospective single-centre analysis of all patients undergoing CRS and HIPEC for CPM between 2007 and 2025. Primary endpoint was overall survival (OS); secondary analyses assessed individual TOO components and associated factors.

**Results:**

Eighty-four patients met inclusion criteria (age 54.1 ± 12.1 years, PCI 5.8 ± 4.5). CC0 resection was achieved in 88.1 %, severe postoperative complications occurred in 34.5 %, and reoperation was required in 20.2 % of patients. Ninety-day mortality was 1.2 %. Complete TOO was achieved in 14.3 % of patients as only 31/84 were recommended adjuvant chemotherapy. Median OS was 39.2 months. Absence of reoperation (p=0.02), and negative lymph node status (p=0.04) were significantly associated with improved OS.

**Conclusions:**

TOO was achieved in <50 % of patients, mainly due to the absence of adjuvant chemotherapy. Absence of reoperation was associated with survival, suggesting its validity as quality indicator. Refinement of TOO definitions, incorporating patient-centred recovery measures, may improve their applicability.

## Introduction

Colorectal cancer (CRC) is the third most frequent malignant disease worldwide and accounts for the second most cancer-related deaths [1]. The oncological outcome depends on cancer stage, as metastatic CRC often affects liver, lung, and peritoneum with poor prognosis [2]. The presence of metastasis proved to have a dismal impact on overall survival, as 5-year survival rates for non-metastatic stage I and II CRC have been reported between 80 and 90 %, for stage III (lymph node positive) between 65 and 85 % and around 20 % for unresected metastasised CRC [3]. Up to 10 % of CRC patients are diagnosed with peritoneal metastasis (PM), one third of them having no other sites of metastasis at diagnosis [4]. While approximately half of PM is detected synchronous at diagnosis, the other half is developed metachronous [2]. Amongst all metastatic CRC patients, PM have the worst prognosis, which can be explained by suboptimal pharmacokinetics, and limited surgical treatment options [5], 6].

Over the past decades, surgical techniques, application modalities of intraperitoneal chemotherapy, chemotherapeutical drug schemes and patient selection have been discussed and steadily improved [7], 8], resulting in clear indications and consensus of international working groups [9], 10]. Indications for cytoreductive surgery (CRS) as part of a multimodal therapeutic concept, have been patients with favourable tumour biology, resectable peritoneal metastasis, absence of other organ metastasis, while the use of hyperthermic intraperitoneal chemotherapy (HIPEC) is debate of currently ongoing clinical trials [11]. Major morbidity (Clavien–Dindo ≥3b), and mortality of these extensive surgical procedures, have been reported around 20 % and between 0 % and 3 %, depending on the extent of disease and surgery, the grade of specialisation of the centre, as well as patient-related factors [12], [13], [14]. CRS procedures count amongst most complex surgeries, as the learning curve was evaluated to be 40 operations to achieve a low major morbidity and 100 operations for the right patient selection in order to reduce open and close procedures, and therefore proctoring contributes to a safer and quicker implementation of new centres [15], [16], [17].

As an effort to decrease morbidity and mortality, and at the same time improving the surgical quality standardisation of treatment modalities and ERAS concepts have been widely developed [18], [19], [20]. Another piece of the puzzle is the implementation of textbook (oncologic) outcomes (TOO), first described by a Dutch consortium in 2012 for colorectal surgery [21]. The intention of textbook outcomes is to provide a standardised, ideal benchmark for surgical and clinical performance by defining a composite measure of optimal results, allowing objective assessment of quality of care and comparison across centres. In 2024, Zohar et al. published the results of a Delphi process defining six textbook oncologic outcome measures for the treatment of patients with pmCRC treated with CRS and HIPEC: absence of unplanned reoperations for 30 postoperative days, absence of severe postoperative complications (Clavien-Dindo ≥III), absence of unplanned readmissions for 30 postoperative days, absence of 90-day postoperative mortality, absence of contraindications for chemotherapy within 12 weeks from operation, achievement of complete cytoreduction (CC0) [22].

Our study aims to validate the clinical relevance of recently published textbook oncologic outcomes by analysing and classifying patients who underwent CRS and HIPEC at a single tertiary centre for surgical oncology.

## Methods

### Study design and variables

This study is a single centre retrospective analysis. All patients who received CRS and HIPEC at Heidelberg University Hospital with pathologically confirmed colorectal cancer were included in this study, which correspondents to a timeframe from 2007 to 2025.

Primary goal of the study was to compare overall survival (OS) between patients that reached textbook oncologic outcome and those who did not. In addition, variables were collected to assess factors potentially influencing oncologic outcomes. The six TOO were defined and collected for each patient: 30-day reoperations, morbidity via the Clavien-Dindo classification, 90-day mortality, 30-day readmissions, CC-score, contraindications for chemotherapy 12 weeks after surgery. The following additional relevant patient data was collected: age, BMI, ASA score, synchronous or metachronous peritoneal disease, tumour location, tumour stage (T, N, and R at resection), tumour biology (grading, RAS and BRAF mutations, microsatellite stability), Peritoneal Cancer Index (PCI), duration of surgery, length of stay, length of Intensive care unit (ICU) treatment, HIPEC drug and concentration, application technique and duration, and indication for adjuvant chemotherapy.

### Patient selection and treatment

According to the German national S3-guidelines and NCCN guidelines for stage IV colorectal cancer, 61 out of 84 patients were initially treated with systemic chemotherapy including a platin/5-U-based combination of chemotherapy. For the indication of this treatment patients were selected during a multidisciplinary team meeting (MDT) specialised on colorectal cancer including specialists from medical oncology, surgical oncology, radiation oncology, radiology, and pathology. Inclusion criteria were as follows: 1) patients between 18 and 85 years old, 2) histological confirmation of PM of CRC or T4 status with high risk for metachronous PM in two cases, 3) resectability estimated by CT or MRI scan. Exclusion criteria were as follows: 1) patient with any other distant metastasis (e.g. liver, lung, bone).

CRS was carried out to achieve complete cytoreduction. To achieve this, patients underwent colorectal resection, peritonectomy procedures (diaphragmatic peritonectomy, parietal peritonectomy, pelvic peritonectomy, omentectomy, and peritonectomy of the small bowel meso). Right sided colonic resections were defined as right colectomy and or transversal colonic resection, while left sided colonic resections were defined as left colectomy, sigmoid, and rectal resections. Complete cytoreduction was defined as absence of visible tumour nodules (CC=0). HIPEC was initially administered via a modified heart-lung machine, followed by a RanD Biotech machine maintaining an intrabdominal temperature of 41 °C–42 °C. The following chemotherapeutic regimen has been used for HIPEC: 15 mg/m^2^ mitomycin C and 15 mg/m^2^ doxorubicin for 90 min (2008–2011); 300 mg/m^2^ oxaliplatin for 30 min (2012–2018); no HIPEC (2019–2022); 15 mg/m^2^ mitomycin C for 60 min (2023–2025).

### Clinical data, survival, and follow-up

Data collection was performed by extraction of surgical data through the hospital information system (i.s.h.med, Oracle Cerner/SAP, USA/Germany). Patient- and disease-related data was collected from archived clinical files. Information on survival, if not documented in the hospital system, was obtained by institutional request to the German residents’ registration office. This study was conducted in accordance with the principles of the Declaration of Helsinki and was approved by the local ethics committee (approval number: S-136/2021). The median follow-up was 20.3 months.

### Statistical analysis

All statistical analyses were conducted using SPSS Statistics version 25 (IBM SPSS, Armonk, NY) or Prism 10 (Graphpad Software, Inc., La Jolla, CA, USA). Continuous descriptive data are given as mean and standard deviation or median and interquartile range. Categorical data are displayed as frequencies and proportions. Univariate analysis of time to event data was performed using log-rank test, including 25 % and 75 % quartiles to compare several groups. Univariate results were visualized by Kaplan–Meier curves, including 95 % confidence interval. Log-rank test was used to compare different variables. A p value of <0.05 was considered statistically significant. For the primary endpoint of the study, patients were categorised into two categories: Those who achieved textbook oncologic outcome and those who did not.

## ⁠Results

**Figure 1:**
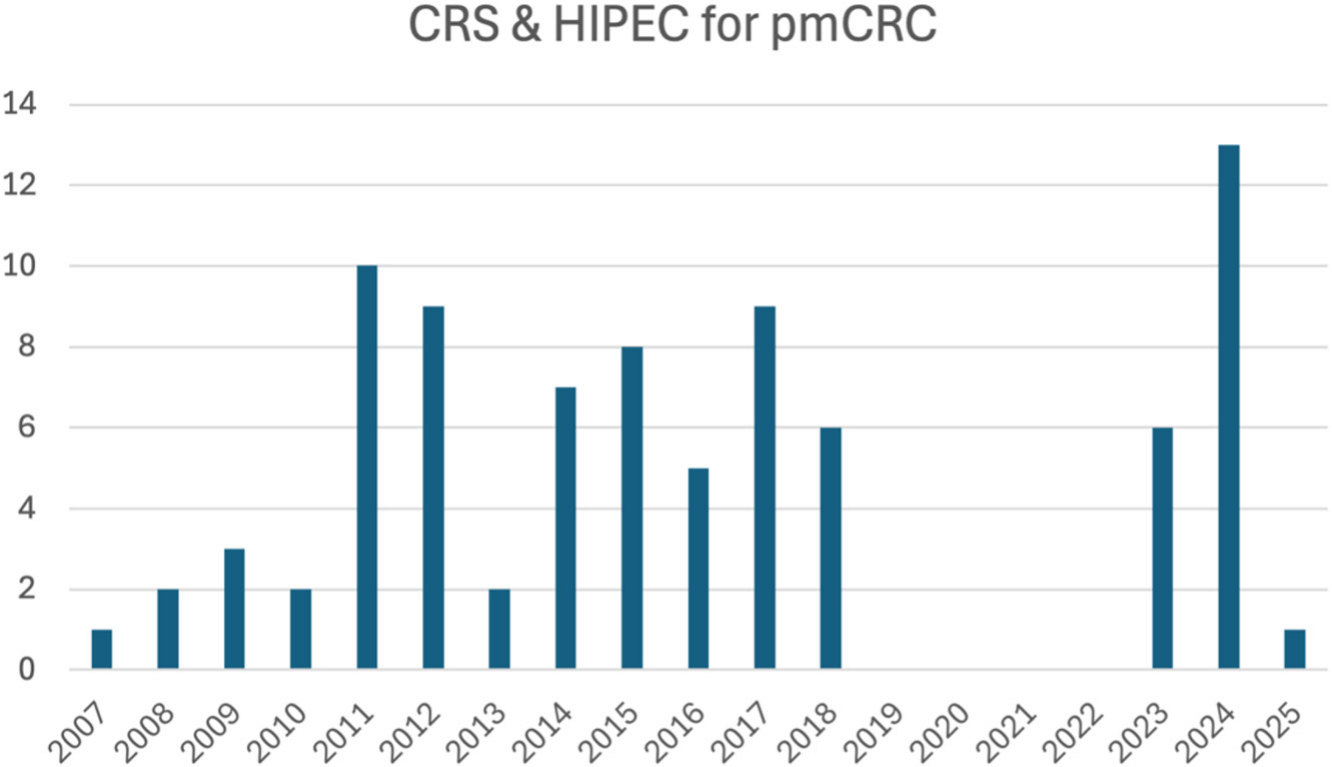
Number of cytoreductive surgery (CRS) and hyperthermic intraperitoneal chemotherapy (HIPEC) per year at Heidelberg University Hospital.

From 2007 until 2025, a total of 302 patients were treated with CRS and HIPEC at our institute, 84 of these due to colorectal peritoneal metastasis (CPM). Throughout 2019 and 2022 HIPEC was not added after CRS (Figure 1). The mean age of patients was 54.1 ± 12.1 years, with a female ratio of 51.2 %, and a mean PCI of 5.8 ± 4.5. Most patients were treated due to metachronous PM (n=50), while 32 patients suffered from synchronous PM. Two patients were prophylactically treated with HIPEC due to T4 primary cancers in the absence of PM. Out of the 50 patients with metachronous PM, six had undergone their primary surgery at Heidelberg University Hospital, whereas 44 patients were referred after primary resection elsewhere. A total of 31 patients were recommended adjuvant systemic chemotherapy during postoperative MDT. Further demographics and clinical characteristics are illustrated in Table 1.

**Table 1:** Demographics from patients treated with cytoreductive surgery and hyperthermic intraperitoneal chemotherapy.

Factor	Total (n=84)	Missing values
Female, %	51.2 (43/84)	0
Age, years	54.1 ± 12.1	0
BMI, kg/m2	27.0 ± 5.8	0
ASA−classification, %		0
I	1.2 (1/84)	
II	36.9 (31/84)	
III	61.9 (52/84)	
IV	0 (0/84)	
Tumor size		1
T 1	0	
T 2	0	
T 3	36.1 (30/83)	
T 4	63.9 (53/83)	
Nodal status		1
N 0	21.7 (18/83)	
N 1	30.1 (25/83)	
N 2	47.0 (39/83)	
N 3	1.2 (1/83)	
Tumor grading		23
G 1	9.8 (6/61)	
G 2	49.2 (30/61)	
G 3	41.0 (25/61)	
PCI score	5.8 ± 4.5	51
Sequence peritoneal metastasis		
Synchronous	38.1 (32/84)	
Metachronous	59.5 (50/84)	
Prophylactic	2.4 (2/84)	
Tumor localization, %		0
Right colon	41.7 (35/84)	
Transversal colon	7.1 (6/84)	
Left colon	31 (26/84)	
Rectum	17.9 (15/84)	
Double location	2.4 (2/84)	
RAS positive	47.9 (34/71)	13
BRAF positive	15.9 (7/44)	40
MSI	9.6 (5/52)	32
Preop chemotherapy	72.6 (61/84)	0

The median length of stay at the ICU was 4 days, and hospital stay 20 days. The mean operative duration was 5.8 ± 2.3 h. HIPEC was mainly performed using a closed technique (83/84), duration and drug varied during the observational period. Major postoperative complications (Clavien-Dindo ≥3b) occurred in 20 patients (23.8 %), while two patients died (2.4 %) during their hospital stay, one of whom died within 90 days after surgery. Further details are illustrated in Table 2.

**Table 2:** Treatment related data from patients treated with cytoreductive surgery and hyperthermic intraperitoneal chemotherapy.

Factor	Total (n=84)	Missing values
Duration operation, hours	5.8 ± 2.3	0
CC status		0
CC0	88.1 (74/84)	
CC1	8.3 (7/84)	
CC2	3.6 (3/84)	
CC3	0 (9/84)	
R-status		32
0	75.0 (39/52)	
1	19.2 (10/52)	
2	5.8 (3/52)	
HIPEC		
Closed technique	98.8 (83/84)	
Simultaneous	84.5 (71/84)	
Drug		1
MMC 15 mg/m^2^	25.3 (21/83)	
Oxaliplatin 300 mg/m^2^	56.6 (47/83)	
MMC/doxorubicin 15 mg/m^2^/15 mg/m^2^	18.1 (15/83)	
Duration		1
25 min	1.2 (1/83)	
30 min	52.4 (44/83)	
60 min	25.0 (21/83)	
90 min	21.4 (18/83)	
Postoperative complication (Clavien-Dindo)		0
0	28.6 (24/84)	
1	9.5 (8/84)	
2	27.4 (23/84)	
3a	10.7 (9/84)	
3b	8.3 (7/84)	
4a	10.7 (9/84)	
4b	2.4 (2/84)	
5	2.4 (2/84)	
Re-operation	20.2 (17/84)	0
Length of ICU, days	4.0 (range: 150)	0
Length of hospital stay, days	20.0 (range: 147)	0

The TOO were achieved as follows: CC0-status in 74 patients (88.1 %), no major postoperative complications in 55 patients (65.5 %), no re-operation in 67 patients (79.8 %), no 30-day readmission in 77 patients (91.7 %), no 90-day mortality in 83 patients (98.8 %), and return to systemic therapy within 12 weeks in 25 out of 26 patients (96.2 %).

Combining all six TOO measures, a total of 12 patients (14.3 %) reached complete textbook outcome, when excluding return to systemic chemotherapy from this analysis, a total of 46 patients (54.8 %) achieved complete textbook outcome.

**Figure 2:**
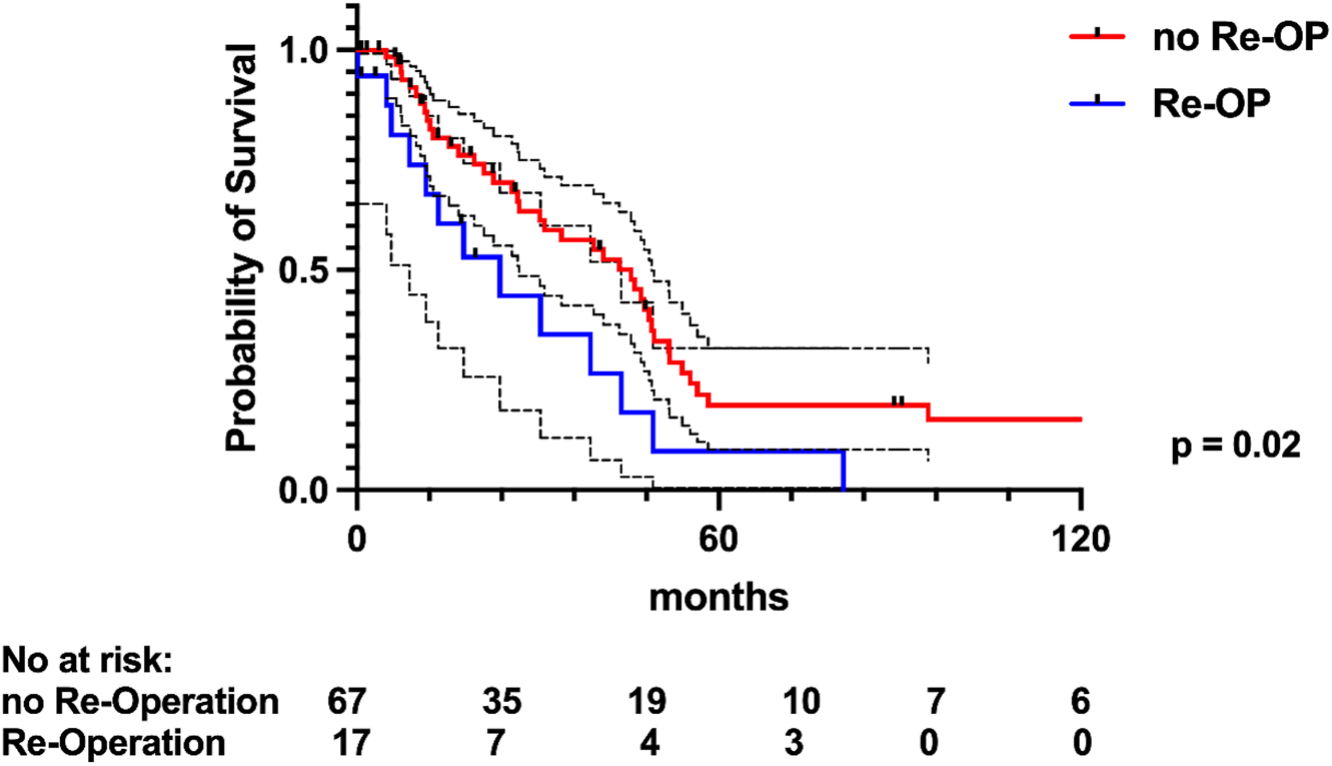
Overall patient survival comparing patients who needed re-operation compared to did not.

The TOO re-operation and 90-day mortality showed significant impact on overall survival in the univariate analysis and are visualised in Table 3 and Figure 2.

**Table 3:** Univariate analysis of factors affecting survival of colorectal cancer patients with peritoneal metastasis after cytoreductive surgery and hyperthermic intraperitoneal chemotherapy; overall survival is illustrated as median with 25% and 75 % quartiles.

Variable	Category	n	Median overall survival [months]	Univariate analysis (p-Value)
Sex	Male Female	41 43	46.0 (35.7–56.3) 30.4 (14.9–45.9)	0.51
Age	≤60 years >60 years	53 31	40.8 (24.3–57.4) 38.7 (13.2–64.3)	0.49
Primary tumor location	Right sided Left sided	41 43	33.9 (20.5–47.3) 45.4 (21.6–69.2)	0.97
T –status	3 4	30 53	26.9 (16.9–36.9) 43.8 (35.5–52.1)	0.31
Lymph node status	Negative Positive	18 65	51.7 (9.0–95.4) 30.4 (17.7–43.1)	**0.04**
R–status	R 0 R 1 or R 2	39 13	33.9 (16.2–51.5) 43.8 (20.7–66.9)	0.68
Tumor grading	G 1 and G 2 G 3	36 25	39.2 (25.5–52.9) 26.6 (20.6–32.7)	0.70
KRAS	Positive Wild type	34 37	33.9 (14.4–73.2) 43.8 (20.5–47.3)	0.27
BRAF	Positive Wild type	7 37	13.5 (4.1–23.0) 45.4 (36.0–54.8)	0.29
Microsatellite	Stable (MSS) Instable (MSI)	47 5	46.0 (21.2–70.8) 22.6 (n.a.)	0.46
Sequence peritoneal metastasis	Synchronous Metachronous	32 50	46.0 (22.6–69.4) 33.9 (17.9–49.9)	0.37
Preop. chemotherapy	Yes	61	31.1 (14.5–47.8)	0.12
	No	23	49.1 (31.4–66.8)	
Treatment year	2007–2015 2016–2024	44 40	40.8 (25.7–56.0) 33.9 (12.5–55.3)	0.34
HIPEC duration	30 min 60–90 min	45 39	38.7 (19.8–57.6) 43.5 (21.6–65.5)	0.49
HIPEC drug	Oxaliplatin MMC based	47 36	40.8 (25.0–56.7) 39.2 (20.0–58.5)	0.46
**Textbook outcome**	Achieved Not achieved	12 14	47.1 (27.8–66.5) 47.6 (16.1–79.0)	0.73
CC status	Achieved Not achieved	74 10	38.7 (21.6–56.0) 46.0 (25.3–66.7)	0.67
Postoperative complication	Achieved Not achieved	55 29	40.8 (23.6–58.2) 30.4 (14.0–46.9)	0.40
Re-operation	Achieved Not achieved	67 17	45.4 (31.6–59.3) 23.7 (7.2–40.1)	**0.02**
30-day readmission	Achieved Not achieved	77 7	31.1 (17.1–45.2) 43.8 (43.2–44.4)	0.76
90-day mortality	Achieved Not achieved	83 1	39.2 (25.8–52.6) 0.1 (n.a.)	**<0.001**
Return to systemic therapy within 12 weeks	Achieved Not achieved	25 1	47.6 (40.3–54.9) 9.8 (n.a.)	**0.002**

Significant p-values (p<0.05) are illustrated in bold.

Detailed descriptions of the textbook oncologic outcomes were as follows:

Patients with CPM received a complete macroscopic resection of PM (CC0) in 74 cases (88.1 %), three patients (3.6 %) could not reach a CC0/CC1 status.

Major postoperative complications (Clavien-Dindo ≥3) occurred in 27 (32.1 %) patients. 24 patients (28.6 %) suffered none, eight patients (9.5 %) had CD grade 1, 23 (27.4 %) grade 2 complications. Grade 3a occurred in nine (10.7 %), 3b in seven (8.3 %) patients. Nine (10.7 %) patients were readmitted to ICU due to grade 4a, and two (2.4 %) due to grade 4b complications.

Two patients (2.4 %) died during their hospital stay, one of them within 90 days after surgery.

A total of 17 patients had to undergo unplanned reoperations. Reasons were anastomotic leakages (n=6), burst abdomen (n=5), perforation of the small bowel (n=2), complications due to the ostomy (n=2), intraabdominal bleedings (n=1), and diagnostic relaparotomy due to sepsis (n=1). In 12 patients (70.6 %), one re-operation was sufficient to resolve the complication, two needed a second operation, while three patients were re-operated between three and five times.

Seven patients (8.3 %) were readmitted to the hospital. Four due to an infection or collection, two due to incomplete bowel obstruction and one due to acute kidney disfunction.

The analysis of the textbook outcome “absence of contraindications for systemic chemotherapy within 12 weeks” was burdensome and of limited value as in 51 of 84 patients, the postoperatively held MDT did not recommend adjuvant systemic chemotherapy. Additionally, patients often continued their adjuvant chemotherapy treatment in an outpatient setting or in another, geographically closer clinic or abroad, without returning to Heidelberg University Hospital, leading to missing data in five out of 31 patients. One of the remaining 26 patients was not fit enough to undergo chemotherapy 12 weeks after surgery.

**Figure 3:**
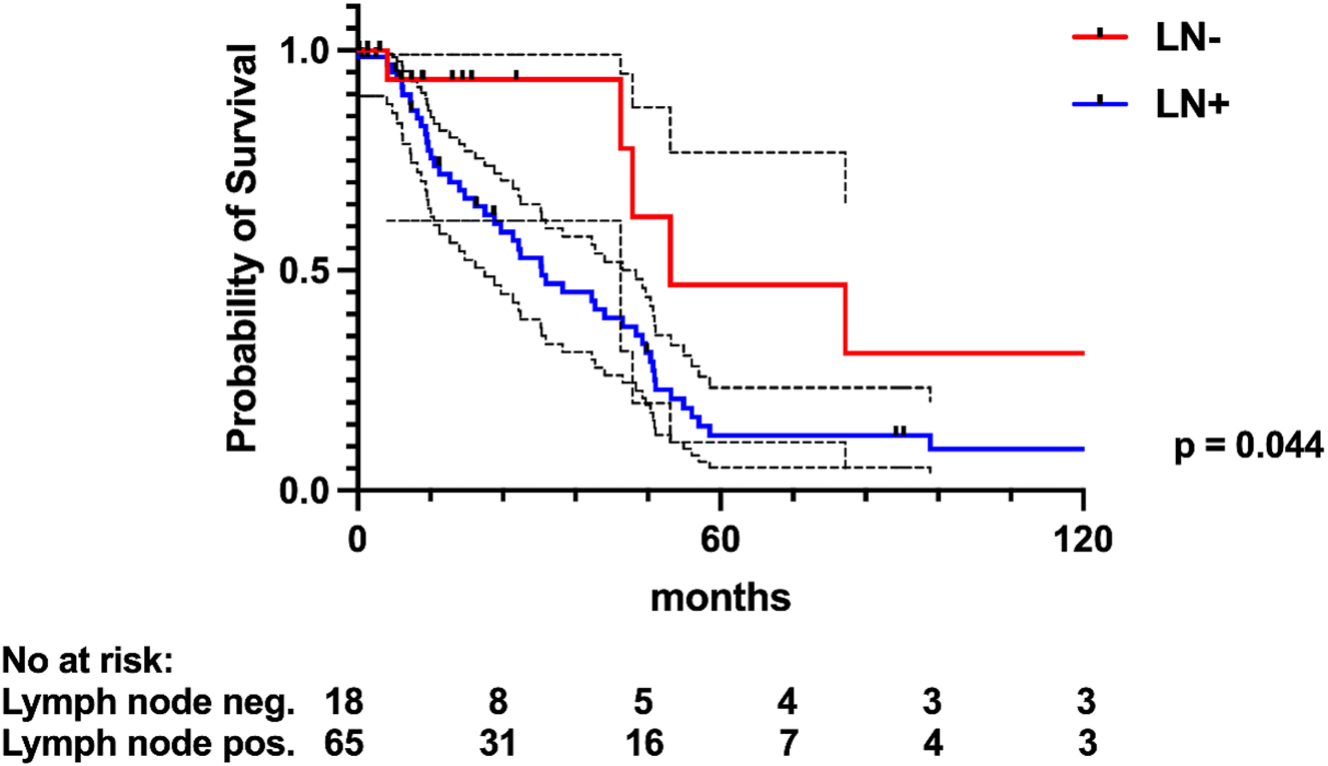
Overall patient survival comparing patients with negative and positive lymph node status.

The median overall survival of the cohort was 39.2 (25.8–52.6) months. The correlation of patient, tumour, and treatment related factors with reaching textbook outcomes did not show any significant correlation (Table 4), Factors associated with survival were negative lymph node status (OS 51.7 vs. 30.4. months, p=0.04) and are illustrated in Figure 3.

**Table 4:** Correlation of patient, tumour, and treatment related factors with achieving textbook outcome.

Factor	Textbook outcome	No textbook outcome	p-Value
Sex			0.48
Male	7	10	
Female	5	4	
Age			0.15
≤60 years	10	8	
>60 years	2	6	
ASA-classification			0.55
I	1	0	
II	7	9	
III	4	5	
Tumor size			0.27
T 3	5	3	
T 4	7	11	
Nodal status			0.35
Negative	0	1	
Positive	12	13	
Tumour grading			0.19
G 1 + 2	3	8	
G 3	6	5	
Resection margin			0.88
R 0	5	6	
R 1 + 2	2	2	
Sequence peritoneal metastasis			0.46
Synchronous	6	9	
Metachronous	6	5	
Tumor localization [%]			0.72
Right sided	6	6	
Left sided	6	8	
RAS			0.86
Wildtype	7	6	
Positive	5	5	
BRAF positive			0.38
Wildtype	8	5	
Positive	1	2	
Microsatellite status			0.41
Stable	7	10	
Instable	0	1	

## ⁠Discussion

Formal TOO was achieved in only 12 out of 84 patients. The main reason was – due to the suggestion of the MDT – the lack of indications for adjuvant chemotherapy (51/84), which led to burdensome interpretation of this TOO as a whole.

### Adjuvant chemotherapy

While there is no doubt about the value of systemic chemotherapy as a palliation in stage IV colorectal cancer patients, there is a lack of evidence regarding the usage of perioperative systemic chemotherapy for patients treated with CRS and HIPEC due to CPM. Two recent systematic reviews evaluated the benefit of adjuvant systemic chemotherapy and both concluded that either there is no benefit or there might be a benefit for adjuvant treatment especially for patients treated with incomplete cytoreductive surgery, which might not entirely match with the definition of the term adjuvant [23], 24]. Based on the lack of evidence, and the fact that nowadays the surgical quality is widespread high and centres master patient selection, CC0 rates – as in our study – from close to 90 % are frequently reported [25], we challenge the value of including returning to systemic chemotherapy within 12 weeks as a relevant TOO. Potential values in the same direction might be: a) return to normal life, such as usual activities or work or b) patient reported recovery (patient related outcome, quality of life).

Univariate survival analysis performing log-rank test revealed four main factors associated with better overall survival: negative lymph node status, re-operation, 90-day mortality, and return to systemic chemotherapy within 12 weeks.

### Lymph node status

In contrast to non-metastatic colorectal cancer, in which lymph node involvement is a significant prognostic factor, decreasing 5-year overall survival from 71.4 % in lymph node negative patients to 8.3 % in lymph node positive patients with a ratio ≥0.7, the evidence in patients with CPM is less robust [26], 27] Amongst many publications, only a few are highlighting lymph node status, such as utilizing N2 vs. N0/1 in a predictive nomogram [28]. The incidence of N0 primary tumours in this study was 21.7 %, which is lower compared to French or Dutch randomised trials reporting incidences between 30 % and 40 % [25], 28], 29].

### Re-operation

Overall, 17 (20.2 %) patients needed to be re-operated, mainly due to anastomotic leakage and burst abdomen, which moderately exceeds the experience from national registries, and recommended rate of 15 % [13]. There is some evidence on the impact of re-operations and especially anastomotic leakages on the long-term oncologic outcome of patients with colorectal cancer, who showed inferior 5-year overall survival [30], 31]. There are several potential explanations such as a delay to adjuvant systemic therapy, a higher local recurrence rate or the creation of an immunosuppressive, pro-inflammatory environment [32], 33]. Potentially, the combination of these factors contributes to a lower 5-year overall survival rate.

The management of complications has become less invasive in high volume centres, especially due to the early detection and the development of interventional radiology. A most recent publication from the Basingstoke group including 1,313 patients illustrated the interventional treatment of 106 (8.0 %) postoperative intraabdominal collections by image guided percutaneous drainage. Even 15 patients with anastomotic leakage could be successfully treated without re-operation, highlighting less invasive options to resolve postoperative complications [34].

### Return to systemic chemotherapy

Systemic chemotherapy is the backbone of treatment in stage IV disease and plays a crucial role as adjuvant therapy especially in patients treated with incomplete cytoreductive surgery [23]. The fact of returning to systemic chemotherapy on time acts as a surrogate parameter of recovery from the extensive cytoreductive procedure and therefore correlates with overall survival. In our study, less than half of the patients were recommended to undergo adjuvant systemic treatment within 6–12 weeks after surgery. Most patients adhered to this recommendation. One patient was physically not able to undergo chemotherapy, while one quarter of those patients were lost to follow-up. With only one patient surely not reaching this TOO, the statistical analysis showed decreased median overall of 9.8 vs. 47.6 months (p=0.002). As already mentioned above, we believe that this item might be subject of rethinking as adjuvant chemotherapy is not standard of care, especially as centres reach CC0 rates of 90 % and above.

### 90-Day mortality

In our study, one patient died withing 90 days after CRS and HIPEC. The analysis on overall survival demonstrated a significant impact comparing 0.1 vs. 39.2 months median survival (p<0.001). This result does not surprise, as postoperative deaths will always be significant to oncological outcomes. It is likely that most well-selected patients who die within 90 days after surgery, pass away due to surgical complications, whether than due to oncologic reasons. The benefit of this measure to determine the effect on overall survival can also be challenged in a mathematical way, as mortality within 90 days will per definition have a negative impact on OS compared to patients that outlive 90 days. Without discussion, postoperative mortality is an important factor in the safety of a treatment and should not exceed 2–3% (Foster et al., [12]).

### Comparison with other published TOO benchmarks in CRS-HIPEC or colorectal surgery

Per today, there is only one analysis of TOO in CRS and HIPEC for patients with CPM published by Zohar et al. 2024. The authors treated a total of 251 patients and met TOO criteria in 60 %. The median overall survival was better for patients meeting TOO reaching 67.5 vs. 44.6 months for patients failing in TOO (p<0.001). While analysing the cohort, there are a few differences regarding the reasons of patients not achieving TOO in our study compared to Zohar et al.

Our study revealed higher rates in postoperative morbidity (Clavien-Dino ≥3) of 32.1 % vs. 19.9 %, and reoperations of 20.2 % vs. 11.6 %. Main reasons for re-operation were anastomotic leakage and burst abdomen, as surgical complications were responsible for the majority of postoperative complications.

The factors of complete cytoreduction, as well as return to systemic chemotherapy were comparable in both studies (88.1 % vs. 92 %; 96.2 % vs. 98.4 %).

Readmission within 30 days showed a difference in comparing the two cohorts with 8.3 % vs. 14.7 %. There are potentially several factors contributing, which could be also related to the differences in health care system, as postoperative recovery differs from country to country, and might be more often continued in outpatient or rehabilitation settings comparing to Germany.

90-day mortality was lower in our study (1.2 %) compared to Zohar at el., which stated 8.4 %. Potential explanations are speculative and could be related to patient and treatment related factors, as well as postoperative complication management and failure to rescue [35]. As there is a lack of precise data from Zohar et al., any further analysis seems not possible.

### Limitations of the study

The major limitation of this study is the retrospective design, where patients were selected, and therapeutic options recommended based on our centre experience in peritoneal surface malignancies. Therefore, selection bias was inevitably a factor in patient enrolment. In addition, the 18-year period, during which substantial changes in surgical techniques, perioperative care, and systemic treatments occurred, contributed to a potential temporal confounding. Another factor was the change of treatment over the years, as negative results of PRODIGE 7 lead to omitting HIPEC during the years 2019–2022. The main factors in patient selection were performance status, PCI, tumour biology and tumour response after preoperative chemotherapy. Finally, our analysis was biased because postoperative systemic chemotherapy was recommended by the multidisciplinary tumour board in only a minority of patients (33 of 84), thereby confounding the analysis of textbook outcomes.

### Future directions

The definition of textbook outcomes is an important step in standardisation of a complex therapeutic option for patients with CPM. Standardisation leads to a better comparison, and a needed benchmarking especially as CRS and HIPEC numbers are rising. Centralisation leads to excellence in therapy, and consequently a better outcome for patients in the multimodal treatment of CPM. As the definition of textbook outcomes is a process, and work in progress, further studies will contribute to shape and potentially improve the definition, as validation is a cornerstone in this process.

## ⁠Conclusion

In this single-centre analysis of 84 patients undergoing CRS and HIPEC for CPM, formal textbook oncologic outcome (TOO) was achieved in fewer than half of patients, largely due to the limited indication for adjuvant systemic chemotherapy in the postoperative setting. While certain TOO components – particularly absence of re-operation and 90-day mortality – showed a clear association with improved overall survival, the inclusion of “return to systemic chemotherapy within 12 weeks” warrants reconsideration given the lack of standardisation and limited evidence for benefit in the modern era of high CC0-rates. Our findings highlight the prognostic value of selected TOO measures, the potential need to refine current definitions, and the importance of ongoing benchmarking to improve quality of care in CRS and HIPEC. Future multicentre prospective studies are essential to validate and optimise TOO criteria, ensuring they reflect meaningful clinical endpoints and patient-centred outcomes (Figure 3).
